# Psychiatric management of fibromyalgia

**DOI:** 10.1192/j.eurpsy.2025.247

**Published:** 2025-08-26

**Authors:** A. J. Krupa

**Affiliations:** Department of Affective Disorders, Jagiellonian University Medical College, Cracow, Poland

## Abstract

**Abstract:**

Fibromyalgia is not a primarily psychogenic disorder, however due to high levels of comorbidity with psychiatric disorders and shared therapeutic targets, psychiatrists can play an important role in the diagnosis and treatment of fibromyalgia.

Fibromyalgia is a chronic pain syndrome affecting around 2-5% of the general population. Fibromyalgia’s core symptom is the persistent generalized pain, its other symptoms include fatigue, stiffness, depressed mood, anxiety and sleep disturbance. Although some psychiatric symptoms are an integral part of fibromyalgia clinical presentation, in a significant part of patients their severity warrants a specific psychiatric diagnosis and management. Available data shows high rates of psychiatric comorbidity in fibromyalgia, with lifetime prevalence reaching 9-33% for anxiety disorders, 16% for post traumatic stress disorder, 63% for depression, 26% for bipolar disorders. Psychiatric comorbidity in fibromyalgia is associated with the severity of fibromyalgia, its impact on patients functioning, quality of life and socioeconomic status. Moreover, the works of our team have also shown that some psychopathological symptoms such as depression, anxiety, anhedonia, chronobiological preferences and circadian rhythm disruptions as well as some psychological traits are linked to lack of response to pharmacological treatment in fibromyalgia.

The current knowledge on fibromyalgia etiopathogenesis is incomplete and does not warrant a comprehensive description. There are no biomarkers or objective tests, which would verify the fibromyalgia diagnosis, which is therefore based primarily on the physician’s history-taking and fulfillment of criteria. In effect, the group of patients diagnosed with fibromyalgia is most likely heterogeneous, regarding the biological basis of its symptoms, clinical presentation and susceptibility to treatment. This also means treatment is symptomatic and characterized by limited effects.

Fibromyalgia management requires a multidisciplinary approach, with emphasis on non-pharmacological interventions such as physical activity, cognitive–behavioral/mindfulness psychotherapy, physical therapies. For a large number of fibromyalgia patients these interventions are not sufficiently effective and there is a need for pharmacotherapy. The use of selective serotonin and noradrenaline reuptake inhibitors (SNRI) and pregabalin, which are commonly used to manage depression and anxiety, is supported by best scientific evidence. Also other drugs used for psychiatric disorders such as amitriptyline, gabapentin, quetiapine or naltrexone (in low doses) were proven useful in fibromyalgia management.

This presentation will sum up current knowledge on psychiatric comorbidity in fibromyalgia and treatments which psychiatrists can offer to fibromyalgia patients.

**Disclosure of Interest:**

None Declared

